# Fenpropathrin, A Pyrethroid Pesticide, Induces Dopaminergic Neurodegeneration in *Drosophila melanogaster*

**DOI:** 10.1155/jt/5460515

**Published:** 2026-07-08

**Authors:** Saba Afsheen, Ahmed Shaney Rehman, Swati Chandra, Mohammad Mumtaz Alam, Suhel Parvez

**Affiliations:** ^1^ Department of Toxicology, School of Chemical and Life Sciences, Jamia Hamdard, New Delhi, 110062, India, jamiahamdard.edu; ^2^ Department of Pharmaceutical Chemistry, School of Pharmaceutical Education and Research, Jamia Hamdard, New Delhi, 110062, India, jamiahamdard.edu

**Keywords:** *Drosophila melanogaster*, fenpropathrin, neurodegeneration, Parkinson’s disease, tyrosine hydroxylase

## Abstract

Parkinson’s disease (PD) is a neurodegenerative condition that typically develops as people age and is characterized by the progressive death of dopaminergic neurons in the substantia nigra area of the midbrain. The etiology of PD includes both genetic and environmental components. Epidemiological studies have shown that pesticide exposure is an important contributory risk factor for PD. Fenpropathrin has been shown to have neurotoxic effects on rodents, but the exact mechanism and its ability to replicate the pathological features associated with PD remain unclear. Therefore, the present study investigated the neurotoxic effects of Fenpropathrin in *D. melanogaster*. Fenpropathrin was added to the food at various concentrations, and adult male flies were allowed to consume it for 96 h, after which the LC_50_ value was determined. The administration of Fenpropathrin resulted in notable impairments in the climbing, jumping, and crawling abilities of adult male flies and larvae, along with a significant dose‐dependent reduction in their lifespan. Moreover, it significantly extended the developmental life cycle of *D. melanogaster*. In addition, Fenpropathrin exposure altered oxidative stress responses through a dose‐dependent biphasic modulation of Nrf2 expression and suppressed the expression of B‐cell lymphoma 2 (Bcl‐2) and tyrosine hydroxylase (TH) expression in the brain tissue of male flies. This resulted in the impairment of dopaminergic neurons and subsequent neuronal death through apoptosis. The results of this study suggest that Fenpropathrin exhibits potent neurotoxicity toward dopaminergic (DA) neurons, implicating it as a notable environmental factor contributing to the risk of PD.

**Highlights**

• Fenpropathrin induced locomotor behavioral dysfunction.
• Fenpropathrin prolonged the developmental life cycle of *D. melanogaster.*
• Fenpropathrin reduced the lifespan of *D. melanogaster.*
• Fenpropathrin altered the expression of Nrf2 in a dose‐dependent manner, indicating an imbalance in oxidative defenses.
• Fenpropathrin reduced the expression of tyrosine hydroxylase and antiapoptotic B‐cell lymphoma 2 in the brain tissue of *D. melanogaster.*

## 1. Introduction

Neurodegenerative disorders (NDDs) such as Parkinson’s disease (PD) and Alzheimer’s disease (AD) have come forth as one of the greatest healthcare dilemmas in the current era [1, 2]. PD ranks as the second most prevalent neurodegenerative condition, impacting more than 6 million individuals globally. It is anticipated that this figure will multiply by 2 by the year 2040 [3]. It is usually characterized by symptoms such as tremors, motor dysfunction, stiffness or slowing of movement, and cognitive deficits. The main neuropathological hallmark feature is the loss of dopaminergic neurons and the formation of Lewy bodies, primarily in the substantia nigra region [4]. NDD creates a financial burden on the U.S. economy in terms of direct healthcare costs, amounting to several billion dollars annually, and societal expenses are expected to rise even more. Unfortunately, despite decades of investigations, the exact pathogenesis of PD is still not clear. As a result, current treatment therapies only provide symptomatic relief, but at present, there is no therapeutic intervention that can mitigate or halt the progression of disease. Moreover, alongside genetic alteration, several epidemiological studies have strongly suggested that occupational exposure to environmental toxins, particularly pesticides, is considered to be a notable risk factor for PD [5–7]. Pyrethroid (Pyr) insecticides have gradually replaced biopesticides and are extensively employed in agricultural practices and veterinary medicine worldwide because they offer a cost‐effective solution and the most efficient method for controlling pest populations and improving agricultural productivity. However, due to increased usage of these pesticides, broad concerns about the potential health risks associated with these substances have been raised [8, 9]. According to Liu et al.[10], pyrethroid insecticides are neurotoxic to both insects and mammals. Prolonged and minimal exposure to pyrethroids can result in persistent ailments and detrimental impacts on the neurological, immunological, cardiovascular, and genetic functions of living organisms, leading to the development of teratogenic, carcinogenic, and mutagenic outcomes [11]. Fenpropathrin (Fen) is an α‐cyano pyrethroid used for crop protection in agriculture as well as for domestic purposes such as controlling mosquitoes, flies, ants, and cockroaches [12, 13]. However, the extensive use of this compound in agricultural practices has raised concerns regarding its potential impact on human health and the environment [14, 15]. It has been found that Fen displays prolonged effects in aquatic environments and has a tendency to be extremely hazardous to fish, leading to high toxicity and bioaccumulation of contaminants in fish [16]. It was found that a man in China who had consumed Fen‐contaminated fish for nearly 6 months was subsequently diagnosed with PD [17]. However, it is important to note that single case studies do not provide sufficient evidence to establish a definitive correlation between Fen exposure and parkinsonism [18]. Despite previous reports that indicated that Fen mediated neurotoxicity in animals [18, 19]. The specific link between Fen exposure history and the onset of PD has remained uncertain, taking into consideration the potential that Fen exposure may lead to lesions in DA neurons and contribute to the development of PD. The broad usage of Fen may have serious public health implications [20]. Considering ethical obligations on animals, *Drosophila melanogaster* offers a robust alternative and has been widely used for investigating molecular facets of toxicology and neurobehavior due to its characteristics such as a short lifecycle, low‐cost maintenance, and absence of ethical concerns [21]. Importantly, *Drosophila* possesses a conserved dopaminergic system and shares approximately 75% of human disease‐related genes, with about 60% homology in genes involved in biological functions [22]. These features, along with its well‐characterized genome and extensive genetic tools, make it an ideal model for exploring the molecular mechanisms of PD. Therefore, we have employed *Drosophila* as an in vivo platform to examine the effect of Fen exposure on behavior, survival, developmental effects, and neurodegeneration, with the specific aim of understanding the relationship between Fen and PD.

## 2. Materials and Methods

### 2.1. Drosophila Stock and Culture

*D. melanogaster* strain (Canton S) flies were cultured on standard medium containing agar, corn meal, sugar, and yeast at 24 ± 1°C. Fenpropathrin (> 98% pure Sigma‐Aldrich) was dissolved in acetone and mixed thoroughly to obtain the desired concentration. Canton‐S, which is a wild‐type strain, was acquired from the Bloomington Drosophila Stock Centre, USA.

### 2.2. Experimental Design

**Figure 1:**
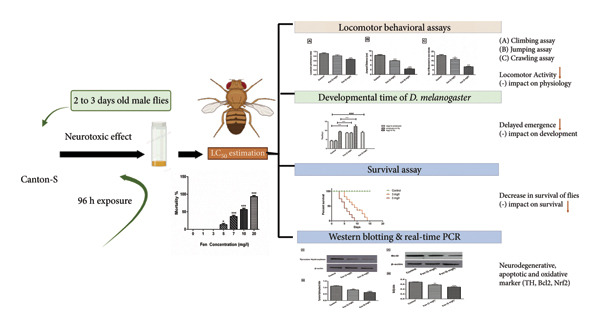
A pictorial illustration of the experimental design and dose selection process.

Figure 1 shows the entire study design, which includes the experimental schedule, dose‐selection technique, and the behavioral, developmental, survival, and molecular assessments.

### 2.3. Drosophila Feeding Regimen and Estimation of the Lethal Concentration (LC_50_)

Fenpropathrin was added to the diet at different concentrations, and ten male flies (2–5 days old) were transferred to a vial for a 96‐h exposure at different Fen concentrations. Adult *Drosophila melanogaster* were anesthetized using diethyl ether for immobilization during observation and handling. A few drops of ether were applied to cotton in a funnel‐fitted etherizer. The fly bottle was gently tapped to move flies near the food surface, then inverted over the etherizer, where the flies became immobilized within seconds. After the exposure period, the total number of dead and live flies was recorded. Based on the mortality rate, it was estimated that the LC_50_ concentration would range from 1 to 20 mg/L. Therefore, to determine the lethal concentration, we selected five different concentrations of Fen (1, 3, 5, 7, 10, and 20 mg/L). A total of 100 male flies (10 flies per vial) were used for every single concentration. Based on the calculated LC_50_ value, we selected working doses of 3 and 5 mg/L. The experiment was repeated in triplicate.

### 2.4. Locomotor Behavioral Assays

#### 2.4.1. Climbing Assay

A negative geotaxis assay or climbing assay was performed as described by Shukla et al. [23] with some modifications. Ten male flies were selected, lightly anaesthetized with ether, and then transferred into an empty glass vial or a 100‐mL glass cylinder. A horizontal line was marked 10 cm above the bottom of the glass cylinder. After recovery, the flies had been acclimated for 1 min. Tap the glass cylinder against a foam pad, and the flies that climbed over a distance of 10 cm (ntop) were scored, while the ones that stayed beneath the mark were also noted (nbot) within a 10‐s time frame. Locomotor performance index (PI) was calculated by applying the formula (1/2 [(ntot + ntop − nbot)/ntot). A total of 10 trials for each control and treated group were taken separately. The experiment was carried out three times independently at 25°C.

#### 2.4.2. Jumping Assay

For evaluation of flight capability, a jumping assay was performed as described by Swank et al. [24]. Male fly wings were first detached, allowed to rest for a certain period of time, and then a single fly was placed on an object around 10 cm high, and then jumping ability was calculated by determining the horizontal distance it could leap from a platform around 10 cm high. The average jump distance of the six longest jumps was measured. A total of 10 trials were taken per fly from the control and treated groups.

#### 2.4.3. Crawling Assay

Larval movement was calculated by following the method of Nichols et al. [25] with some modifications. Third‐instar larvae were selected from the control as well as the Fen‐treated groups, washed in PBS buffer, and then placed in a glass dissection dish well containing a diluted yeast solution. After allowing the larvae to adjust to their surroundings for 5 min, we measured the total number of peristalsis contractions—one contraction equals a full anterior‐to‐posterior movement that the larvae made in a minute.

### 2.5. Survival Assay

Newly eclosed male files were collected and transferred to vials (10 flies per tube) containing normal food and treated food mixed with the desired concentration of Fen. The number of dead flies was counted every third day [26], and survivors were transferred to fresh food until all the flies were dead.

### 2.6. Assay of Preadult Development Period

Synchronized laid eggs were collected from food plates and transferred to treated as well as control groups, which were examined daily. Total time was recorded separately from egg to prepupa, prepupa to fly, and egg to fly. A total of five experimental vials were used for each experimental group. Twenty eggs per vial were used for a total of 100 eggs for one group. The experiment was repeated three times.

### 2.7. Real‐Time PCR

Total RNA was extracted from the heads of approximately 100 *Drosophila melanogaster* using TRIzol reagent (Invitrogen), following the manufacturer’s instructions. RNA concentration and purity were assessed using a NanoDrop spectrophotometer, and RNA integrity was confirmed by agarose gel electrophoresis. For cDNA synthesis, 1 μg of total RNA from each experimental group was reverse‐transcribed using the cDNA Synthesis Kit (Thermo Scientific, USA), according to the manufacturer’s protocol. Quantitative real‐time PCR was performed using SYBR Green chemistry on a Bio‐Rad real‐time PCR system. Each reaction was carried out in triplicate under a standard three‐step cycling protocol. Primer sequences were designed using Primer3 software, and relative gene expression was calculated using the 2^−ΔΔCt^ method. Quantitative PCR was performed as previously described by Naz et al. [27]. Primer sequences utilized in this study are as follows (Table 1).

**Table 1:** The primer sequences for the genes studied in *Drosophila melanogaster*.

Genes	Forward sequences	Reverse sequences
GAPDH	ATT TCC GAT CTT CGA CAT GG	GAA AAA GCG GCA GTC GTA AT
Nrf2	TTG GGC TTC AGT TTG G	ATC CGA GGA CTT GGT CT

### 2.8. Western Blotting

For protein sample preparation, 200 fly heads were first frozen in liquid nitrogen and then homogenized using a micro‐pestle in ice‐cold RIPA lysis buffer (20 mM Tris‐HCl [pH 7.5], 150 mM NaCl, 1 mM Na2 EDTA, 1 mM EGTA) in the presence of protease inhibitor cocktail (Abbkine). Homogenates were incubated on ice for 30 min with occasional vortexing to ensure complete lysis. The lysate was then centrifuged at 4°C for 10 min at 10,000 rpm, and the supernatant containing total soluble protein was carefully collected. Protein concentration was measured using the Bradford assay. Approximately 30–50 μg of protein per sample was resolved by 6%–15% SDS‐PAGE gel electrophoresis and subsequently shifted to nitrocellulose membrane. To prevent nonspecific antibody binding, membranes were blocked in a 5% skimmed milk solution (Millipore) in PBST for 1 h at room temperature. After blocking, the membrane was incubated with primary antibody Bcl_2_ (G‐Bioscience); anti‐TH (AB 152 Merck) in 1:1000 dilutions overnight at 4°C. The next day, after washing the membrane with PBST, incubate the membrane with secondary antibody (anti‐rabbit Invitrogen, anti‐mouse Abbkine) for 1 h at 24°C–25°C, 1:1000 dilution. Following additional PBST washes, protein bands were visualized using an enhanced chemiluminescent (ECL) detection reagent. Images were captured and analyzed using the ChemiDoc MP imaging system (BIO‐RAD).

### 2.9. Statistical Analysis

The GraphPad Prism software (Version 6) was utilized for conducting the data analysis. In order to assess the statistical significance of differences between multiple groups, an analysis of variance (ANOVA) can be conducted, followed by a Tukey test. The data values were presented in the form of mean ± SEM. Statistical significance was determined by considering *p*‐values less than 0.05. Effect sizes were determined using G∗Power analysis based on previously published studies and pilot data [28]. For ANOVA, effect sizes of 0.70 and 0.25 were obtained, with an *α* error of 0.05 and a power of 95%. Accordingly, a sample size ranging from *n* = 6 to 10 for adult flies and *n* = 80 to 100 for eggs was used to evaluate statistical significance (Supporting Table 1A, 1B). No outlier tests were performed, and no data points were excluded from the analysis.

## 3. Results

### 3.1. Fen and Estimation of LC_50_

**Figure 2:**
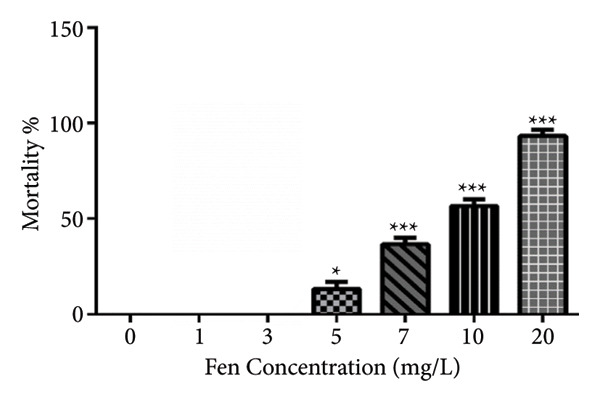
Representative graph showing the effect of Fen on *D. melanogaster* wild‐type (Canton S) adult male flies. The graph displays the mortality rate of five different concentrations of Fen (1, 3, 5, 7, 10, and 20 mg/L) on *D. melanogaster* male adults after 96 h of treatment. No mortality was observed in 0, 1, and 3 mg/L dose during the 96‐h period. The significance is assigned as ^∗∗∗^ *p* < 0.001 and ^∗^ *p* < 0.05 compared with the unexposed control. The data are displayed as the mean ± SEM of three separate experiments (*n* = 100 per group) and were analyzed using one‐way ANOVA, then followed by Tukey’s multiple‐comparison test.

*Drosophila* male adults were subjected to varying concentrations of Fen, ranging from the lowest to the highest dose, for 96 h. Based on the mortality rate of flies, it was predicted that the LC_50_ would be in the range of 1–20 mg/L. Thus, in order to determine the LC_50_, six different concentrations of Fen (1, 3, 5, 7, 10, and 20 mg/L) were selected based on the observed mortality rates of the flies. No instances of fly mortality were observed during the 96‐h period in both the control group and the group exposed to the lowest concentration of Fen, which was 1 and 3 mg/L. Nevertheless, an instance of mortality was noted after a duration of 96‐h time frame, thereby suggesting that a prolonged period of exposure is necessary to detect fatal effects in low doses of Fen. According to the mortality data as outlined in the graph (Figure 2), the estimated LC_50_ value was determined to be 10.23 mg/L, which caused the deaths of 50% of adults. Thus, we opted to employ values that were approximately half and one‐fifth of the LC_50_. The statistical analysis revealed a significant difference in the mortality rate of flies across varying concentrations of Fen, *p* < 0.001, *p* < 0.05, respectively, when compared to the unexposed control group.

### 3.2. Exposure to Fen Impairs Locomotor Activity

The results of the climbing assay indicated a reduction in locomotor activity in Fen‐treated adults. The locomotory PI was observed to have a significant reduction at a concentration of 5 mg/L in Fen‐treated vials as compared to unexposed control flies (*p* < 0.001), as depicted in Figure 3(a). However, no significant delay in climbing ability was observed in the 3 mg/L Fen‐treated adults when compared to the control group. In our present study, we excluded female flies and only used male flies, as female flies displayed different feeding patterns.

Figure 3Effect of Fen on (a) climbing, (b) jumping, and (c) crawling performance of *D. melanogaster* wild type (Canton S) adult male flies. The significance is assigned as ^∗∗∗^ *p* < 0.001, ^∗^ *p* < 0.05 compared with the unexposed control. The data are displayed as the mean ± SEM of three separate experiments (*n* = 6–10 per group) and were analyzed using one‐way ANOVA, then followed by Tukey’s multiple‐comparison test.

**Figure:**
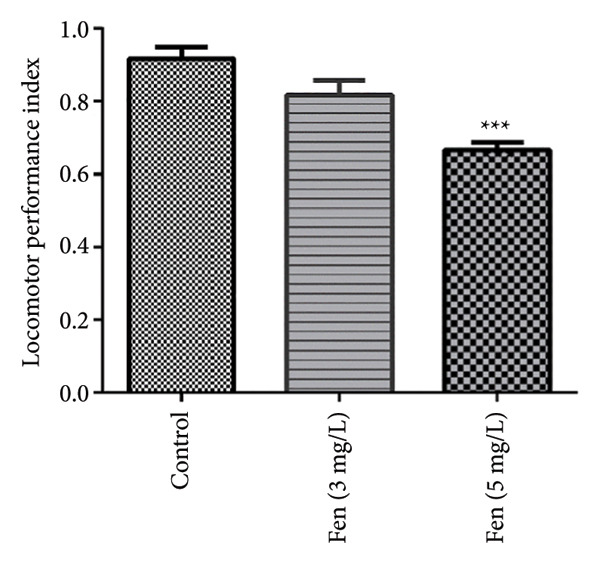
(a)

**Figure:**
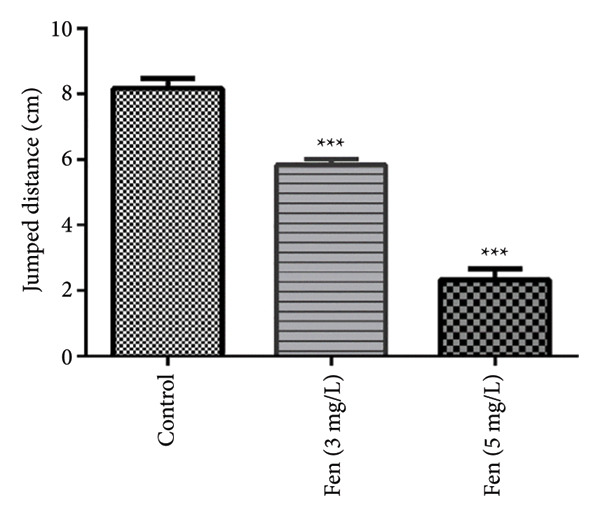
(b)

**Figure:**
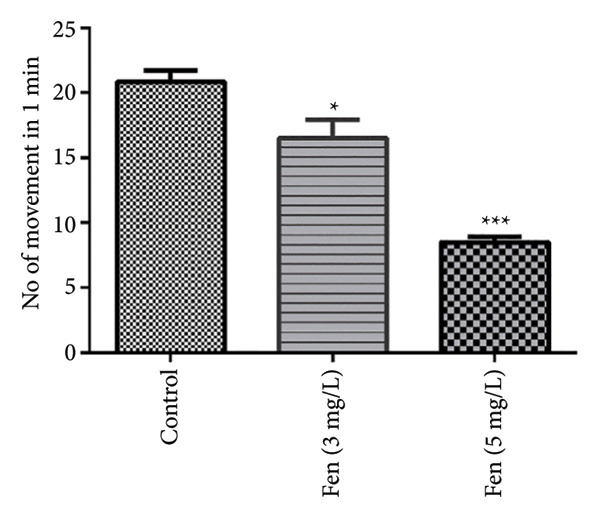
(c)

### 3.3. Exposure to Fen Impairs Jumping Performance

To evaluate the impact of Fen on the jumping behavior of adult male flies, we performed a jumping assay. The study findings indicate that the groups administered with varying concentrations of Fen exhibited a decline in jumping performance when compared to the control group. The findings presented in Figure 3(b) demonstrate a noteworthy decrease in jumping behavior at doses of 5 and 3 mg/L of Fen, as compared to the control or untreated flies (*p* < 0.001).

### 3.4. Effect of Fen Exposure on the Third Larval Crawling Assay

The results depicted in Figure 3(c) displayed defective body wall contractions in the groups treated with Fen as compared to the control group that was not exposed to the treatment. The study observed a noteworthy decrease in body wall contraction in the group treated with 5 mg/L Fen (*p* < 0.001) and 3 mg/L (*p* < 0.05) when compared to the control group that was not exposed to the treatment.

### 3.5. Effect of Fen on Developmental Time Period

**Figure 4:**
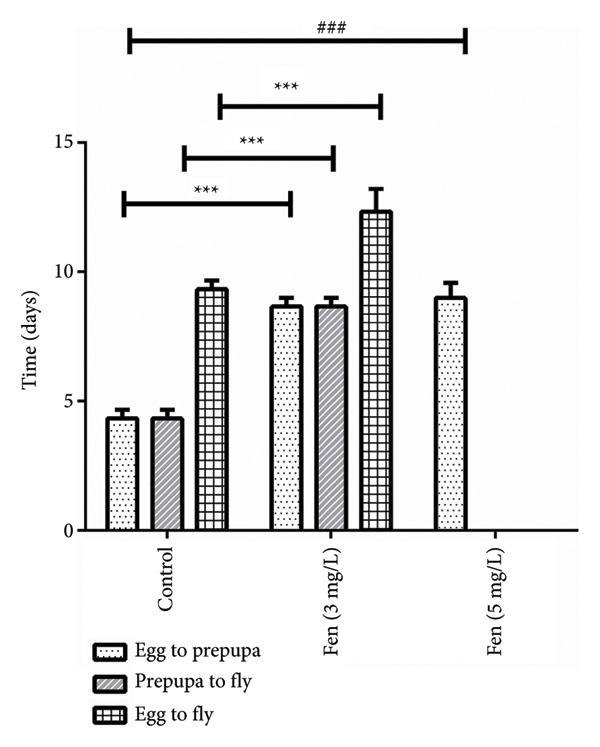
The development time of *D. melanogaster* wild type (Canton S) in different stages after exposing to different concentrations of Fen. The time of the egg‐prepupa was prolonged in the 3 and 5 mg/L treatment group. There was a significant change in the time of the prepupa‐fly in 3 mg/L but no prepupa‐fly development in 5 mg/L. In general, Fen treatment will prolong the developmental cycle of *D. melanogaster*. Statistical significance is ascribed as ^∗∗∗^ *p* < 0.001 for egg to prepupa; prepupa to fly; egg to fly (3 mg/L vs. control), ^###^ *p* < 0.001 for egg to prepupa (5 mg/L vs. control). Data are presented here as mean ± SEM of three separate experiments (*n* = 80–100 eggs per group) and analyzed using one‐way ANOVA, then followed by Tukey’s multiple‐comparison test.

To further evaluate the effects of Fen on the developmental timeline of *Drosophila*, varying concentrations of Fen were administered to *Drosophila* eggs. The duration of the egg‐prepupa developmental stage was observed to be significantly extended in the groups treated with 3 and 5 mg/L concentrations (*p* < 0.001) compared with the unexposed group (Figure 4). A significant alteration in the prepupa‐fly duration was observed in the 3 mg/L group (*p* < 0.001) compared with the control group, while no progression from prepupa‐fly to egg‐fly was evident in the 5 mg/L group.

### 3.6. Exposure to Fen Decreases the Life Span of Drosophila

**Figure 5:**
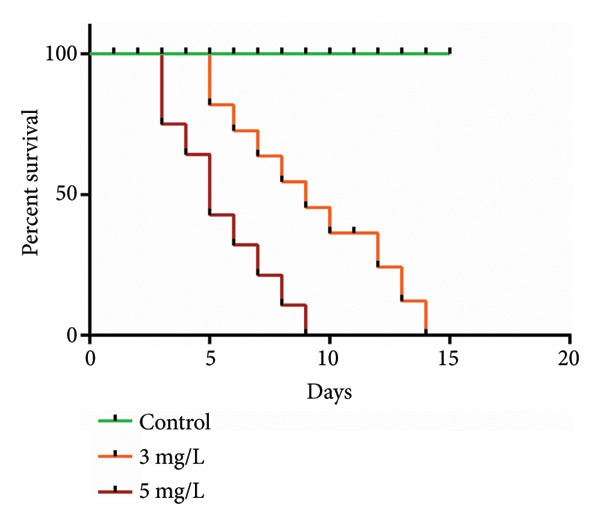
Effect of fen on survival *D. melanogaster* wild type (Canton S) male adults under different doses of Fen. Two‐day‐old male flies were reared in 3 and 5 mg/L (10 flies per vial) for the lifespan till the last fly died. Fen‐containing food vial was changed on every alternate day. The significance is assigned as ^∗∗∗^ *p* < 0.001, compared with the unexposed control. Data are presented here as mean ± SEM of three independent experiments (*n* = 10 per group) and were analyzed using one‐way ANOVA, followed by Tukey’s multiple‐comparison test.

To further examine the Fen effect, we performed a survival assay. The control male flies showed 100% survival for 15 days, but male flies exposed to specified doses of Fen showed a significant decrease in survival rate compared to untreated control male flies. The results obtained from the 5 mg/L Fen–treated group indicate 40% survival on the 6th day and 80% survival in the 3 mg/L Fen–treated group on the 6th day (Figure 5). In the general 5 mg/L group, all the flies survived nearly to the age of 9 days, compared to 14 days of survival in the 3 mg/L group.

### 3.7. Fenpropathrin Exposure Alters Nrf2 Expression in a Dose‐Dependent Manner

**Figure 6:**
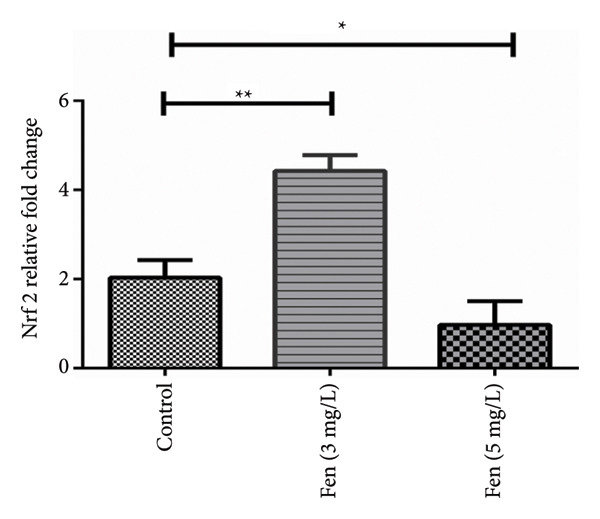
Effect of fenpropathrin on Nrf2 expression in drosophila. Quantitative real‐time PCR analysis showing Nrf2 mRNA levels after exposure to 3 and 5 mg/L Fenpropathrin. Nrf2 expression increased at 3 mg/L but decreased at 5 mg/L compared to the control. Data are presented as mean ± SEM (*n* = 3). The significance is assigned as ^∗∗^ *p* < 0.01 and ^∗^ *p* < 0.05 compared with the unexposed control.

Quantitative real‐time PCR analysis revealed that Nrf2 expression was significantly upregulated in the 3 mg/L Fen–treated group compared to the control, indicating activation of the antioxidant response pathway under moderate stress. However, in the 5 mg/L Fen treatment group, Nrf2 expression was reduced relative to the control (Figure 6), suggesting a possible suppression of the protective response under higher toxic stress, potentially due to cellular damage or regulatory feedback failure.

### 3.8. Fen Exposure Induces Dopaminergic Neurons Loss in Exposed Adult Male Flies

The conversion of L‐dopa into dopamine in dopaminergic neurons is facilitated by the enzymatic activity of tyrosine hydroxylase (TH) [29]. Therefore, the toxic effects of Fen on dopaminergic neurons were evaluated by using western blot analysis, to measure the expression of TH in control as well as Fen‐treated groups (Figure 7). The proposed result shows that the levels of TH expression in the brain tissue were significantly decreased at respective doses of 5 and 3 mg/L of Fen as compared to control or untreated flies (*p* < 0.001).

Figure 7Effect of fen on tyrosine hydroxylase expression in brain tissue of *D. melanogaster* wild type (Canton S) male adults. Expression of Tyrosine Hydroxylase was analyzed by western blotting (a). Densitometric data presented are shown after normalization with loading control β‐Actin (b). Graph (right side) shows quantitative analysis of protein‐band intensity, Fen reduced the expression of tyrosine hydroxylase protein in the 5 and 3 mg/L groups and significance is assigned ^∗∗∗^ *p* < 0.001 vs unexposed control. Data are represented as mean ± SEM (*n* = 3).

**Figure:**
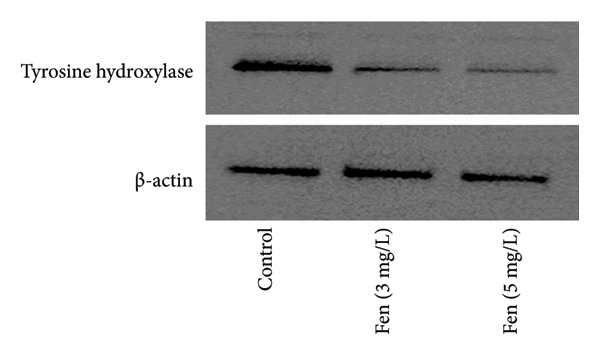
(a)

**Figure:**
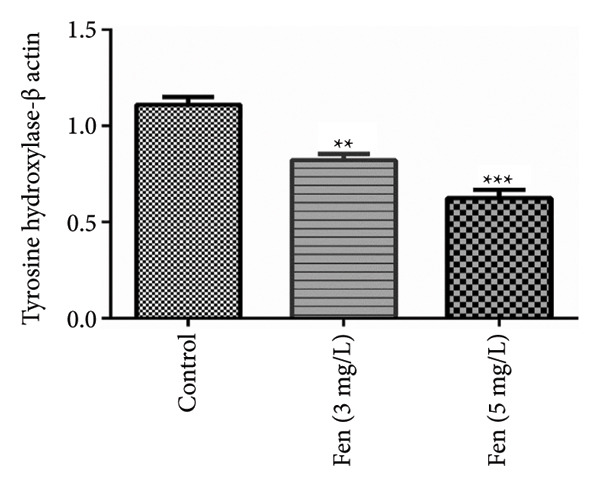
(b)

### 3.9. Fen Exposure Reduces Bcl 2 Expression in Exposed Adult Male Flies

In mammals, Bcl 2 proteins serve as the primary regulators of programmed cell death, and the apoptotic pathway of *Drosophila* exhibits molecular conservation with that of mammals [30]. Therefore, we analyzed the expression of the antiapoptotic Bcl2 protein (Figure 8). According to the study’s findings, western blot analysis showed that the administration of 5 mg/L of Fen caused a notable decrease in the expression of Bcl 2. This effect was observed to be more pronounced in comparison to both 3 mg/L of Fen and untreated control flies (*p* < 0.001).

Figure 8Effect of fen on Bcl2 expression in brain tissue of *D. melanogaster* wild type (Canton S) male adults. Expression of Bcl2 was examined by western blotting (a). Densitometric data are shown after normalization with loading control β‐Actin (b). Protein‐band intensity quantitative analysis (right panel), Fen reduced the expression of Bcl2 protein in the 5 and 3 mg/L groups and significance is assigned ^∗∗∗^ *p* < 0.001^∗∗^ *p* < 0.01 vs unexposed control. Data are represented as mean ± SEM (*n* = 3).

**Figure:**
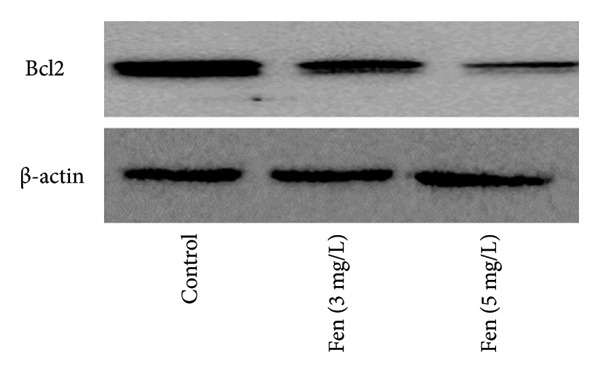
(a)

**Figure:**
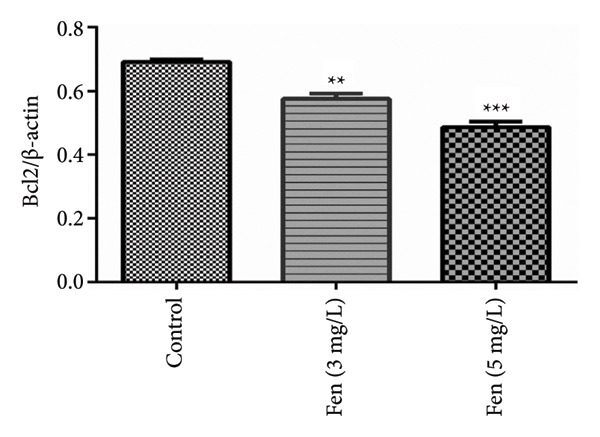
(b)

## 4. Discussion

Pyrethroids are becoming more commonly utilized as pesticides, and their extensive presence in the environment has raised significant concerns regarding their toxicity. Due to their high lipophilicity, these compounds easily penetrate the blood–brain barrier [31]. Pyrethroid exposure is primarily associated with the risk of developing progressive neurodegenerative diseases [32]. In the investigations conducted by Singh et al. [33, 34], it was found that extended exposure to a moderate pyrethroid dose could induce neurological problems. Upon oral administration to rats, pyrethroids exhibit a slow elimination pattern from both blood and nervous tissues. In CNS tissues, the peak concentrations and elimination half‐lives were notably higher compared to those in the plasma [35]. This implies that recurrent exposure to pyrethroids may promote accumulation processes, heightening the susceptibility of animals to neurodegeneration, particularly affecting dopaminergic neurons [34]. *Drosophila melanogaster* is widely regarded as a critical and highly suitable model organism in neurotoxicology research [36], largely due to its well‐characterized nervous system, conserved dopaminergic pathways, ease of genetic manipulation, short life cycle, and the high degree of conservation in molecular and cellular mechanisms of neurodegeneration [37]. Additionally, *Drosophila* provides a cost‐effective, ethically uncomplicated, and time‐efficient platform to investigate neurotoxic mechanisms and perform high‐throughput behavioral and genetic screenings. These features make it an ideal model for exploring fundamental neurobiological questions and screening for potential neuroprotective compounds. Thus, for the purpose of this study, we chose to employ *D. melanogaster* as the model organism to investigate the toxic effects caused by the exposure of *Drosophila* adult organisms to the pyrethroid Fen, which showed a notable degree of toxicity toward *Drosophila* adults, as evidenced by a 96‐h LC_50_ value of 10.23 mg/L. Similarly, it has been reported that pyrethroids such as deltamethrin exhibit moderate to high levels of toxicity in adult *Drosophila*. The 96‐h LC_50_ values for deltamethrin have been documented to be 4.8673 mg/L, respectively, by Aljedan and Dalal [38]. Another study on the pyrethroid cypermethrin revealed that the LC_50_ value was within a range of 80–110 mg/L [39]. In line with the previous report, different insecticides of the pyrethroid class exhibit different toxicity. Our data suggest that Fen exhibits moderate toxicity when compared to cypermethrin and deltamethrin pyrethroids. In earlier studies, the etiology of PD has been linked to reduced lifespan, motor neuron dysfunction, and muscular weakness [40]. Therefore, the current study examined the effect of Fen on various behavioral assays. In the climbing assay, we noticed a delay in the loss of climbing ability in the Fen‐treated group. Similar findings were observed when subacute Fen poisoning decreased locomotor activity at all testing stages in a dose‐proportionate manner [41]. Likewise, a significant reduction in jumping ability was observed in both Fen‐exposed groups, which is in line with the previous study in which Fen‐injected mice showed similar kinds of behavioral deficits [42]. Consequently, we then looked into the effect of Fen on the neuronal activity of third‐instar larvae by performing a larval crawling assay. Fen‐treated larvae showed significant defective body wall contractions compared to the control group, indicating Fen damages the neurons of larvae. Previous studies on the *Drosophila* model of ALS and another study on exposure to rotenone observed a similar kind of larval crawling defect, which is consistent with the current study [43, 44]. The observed defective body wall contraction might be due to the effect of Fen exposure, which causes a slow and permanent decrease in the activity of the neurotransmitter dopamine that regulates locomotor activity in insects. Earlier studies on the impact of various ambient chemicals on various species noted a diminished or delayed appearance of organisms [45, 46]. Likewise, our findings demonstrated that the detrimental effect of Fen on the developmental time of *D. melanogaster* at different stages was apparent through a postponed emergence, which might be due to the adverse effect of Fen on the development of the organism as environmental and genetic factors may play a role in such a developmental transformation. The somatic effects of the chemical in the test media may have contributed to the lengthening of developmental time [47]. To further check the effect of Fen on the lifespan of *D. melanogaster,* we observed a significant reduction in the lifespan of the 5‐ and 3 mg/L‐treated groups compared to control flies. These observations were also supported by previous findings on the effect of Fen lifespan on worker bees, *Apis mellifera* insecta [48]. Pesticides, particularly pyrethroids such as Fen, are known to induce the generation of ROS, leading to oxidative stress by disrupting the cellular redox balance. This oxidative stress plays a pivotal role in the pathogenesis of NDDs, particularly AD and PD [42, 49, 50]. To further elucidate the mechanism, we assessed the expression of Nrf2, a master transcriptional regulator of the oxidative stress response. Over the past decade, numerous studies have established Nrf2 as a central regulator of the cellular antioxidant response, playing a critical role in protecting against oxidative stress [51]. Thus, Nrf2 is recognized as playing an indispensable role in the clearance of reactive oxygen species (ROS). Due to its pivotal role in regulating oxidative stress, we measured Nrf2 expression and found that 3 mg/L of Fen upregulated Nrf2 expression, reflecting a compensatory antioxidant response to moderate stress. However, at 5 mg/L, Nrf2 expression was suppressed, suggesting that overwhelming oxidative damage may inhibit this protective pathway. This biphasic response implicates Nrf2 dysregulation as a central event in Fen‐induced neurotoxicity [52]. Given that dopaminergic neurons are particularly vulnerable to oxidative stress, we investigated TH, the rate‐limiting enzyme in dopamine synthesis and a sensitive marker of dopaminergic neuron integrity [53]. The disruption of the nigrostriatal dopaminergic pathway along with lowered TH activity is observed in the brains of PD patients and in animal models, which is symptomatic of the etiology of the illness [29]. Furthermore, White et al.[54] reported that PD patients’ decreased ability to control their movements is mostly caused by a lack of dopamine, a vital neurotransmitter required for the healthy operation of the body’s motor system. Dopamine synthesis is preserved in both humans and *Drosophila*, and there are separate dopamine clusters that relate to locomotor control, showing many similarities to humans. Previous research on Fen resulted in decreased TH expression in rats, which was accompanied by impaired locomotion [42]. To the best of our knowledge, no studies have been conducted on the impact of Fen on TH expression in *D. melanogaster*. In order to find a correlation between TH expression levels and the observed impaired motor function, we employed the western blotting technique to assess TH expression levels. The results obtained from the present study show that the TH expression levels in brain tissue of D. melanogaster were significantly reduced at doses of 5 and 3 mg/L of Fen. Thus, our findings are consistent with previous studies that Fen resulted in impaired locomotor activity along with an observed reduced level of TH, which is due to the death of dopaminergic neurons.

The loss of nigral dopaminergic neurons in PD is believed to be mostly attributed to apoptosis [55]. In vivo and in vitro, overexpression of Bcl‐2 prevents the death of different neuron types brought on by neurotoxins and other stressors [56]. Prior research conducted in our lab found that the rotenone group also showed decreased expression of the antiapoptotic Bcl 2 protein [57]. In line with the previous study, this study showed significantly reduced expression in the *Drosophila* brain following the administration of 5 mg/L of Fen and 3 mg/L of Fen compared to untreated control flies, indicating enhanced apoptosis in treated groups. Fen exposure led to a significant reduction in Bcl‐2 expression, reinforcing the idea that Fen promotes apoptosis of dopaminergic neurons. The observed downregulation of Nrf2, Bcl‐2, and TH indicates a likely involvement of oxidative stress and apoptotic pathways in Fen‐induced dopaminergic neurodegeneration and associated behavioral impairments, while *Drosophila melanogaster* serves as a valuable model organism due to its conserved genetic pathways, short life cycle, and ease of genetic manipulation, certain limitations must be acknowledged. The simplicity of its nervous system compared to mammals may not fully capture the complexity of human neurodegenerative processes. Although dopaminergic signaling and motor behaviors are conserved, the lack of higher‐order brain functions restricts the translational relevance of certain behavioral outcomes. Therefore, complementary studies using mammalian models are essential to confirm the broader applicability of the findings and to better understand the human relevance of Fen‐induced neurotoxicity.

## 5. Conclusion

The current study evaluated the neurotoxic effect of Fen on the development and behavior of *Drosophila melanogaster*. Fen exposure‐induced behavioral deficits reduced the survival of flies and adversely impacted the developmental lifecycle of *D. melanogaster.* Additionally, Fen treatment resulted in decreased expression of TH and the antiapoptotic protein Bcl‐2, indicating damage to dopaminergic neurons and enhanced apoptotic activity. Notably, Fen exposure also dysregulated Nrf2 expression in a dose‐dependent manner, reflecting disrupted oxidative stress dysregulation. Collectively, these results suggest that Fen is a potential neurotoxin that induces PD‐like symptoms in flies.

To the best of our knowledge, this is the first study demonstrating the neurotoxic effects of Fen on *D. melanogaster*. These results support further investigation into Fen as a potent dopaminergic neurotoxin and a possible environmental risk factor for PD. However, additional research is warranted to elucidate the precise mechanisms by which Fen induces neurodegeneration and PD‐like pathology.

## Ethics Statement

The authors have nothing to report.

## Conflicts of Interest

The authors declare no conflicts of interest.

## Author Contributions

Saba Afsheen contributed to conceptualization investigation, methodology, software, data curation, formal analysis, inspection, resources, validation, writing–original draft, and writing–review and editing. Ahmed Shaney Rehman contributed to software, investigation, and formal analysis. Swati Chandra contributed to software, investigation, and data management. Mohammad Mumtaz Alam performed supervision and writing–review and editing. Suhel Parvez carried out supervision, visualization, resources, writing–review & editing, project administration, funding acquisition, conceptualization, investigation, methodology, software, data curation, formal analysis, validation, writing–original draft, and writing–review and editing.

## Funding

This work was supported by the Fund for Improvement of S&T Infrastructure (FIST) Grant No. SR/FST/LS‐I/2017/05[C] to the Department of Toxicology and the Promotion of University Research and Scientific Excellence (PURSE) Grant No. SR/PURSE Phase 2/39[C] from the Department of Science and Technology (DST), Government of India (to Jamia Hamdard). Saba Afsheen was supported by the Senior Research Fellowship from the Indian Council of Medical Research (ICMR), Government of India (No. AS/JH‐16‐147/2023). Swati Chandra was supported by the HRD Scientist scheme from Department of Health Research (DHR), Government of India (No. 2020‐1318).

## Supporting Information

The supporting files contain G∗Power‐derived sample size justifications for each experiment. We determined the required amount of adult *Drosophila melanogaster* for behavioral and survival assessments, as well as the necessary number of eggs for each developmental phase.

## Supporting information


**Supporting Information** Additional supporting information can be found online in the Supporting Information section.

## Data Availability

Data generated and examined in this study can be obtained from the corresponding author upon reasonable request.
