# Supplementary material for: Fenpropathrin, A Pyrethroid Pesticide, Induces Dopaminergic Neurodegeneration in Drosophila melanogaster
Source: J Toxicol. 2026 Jul 8;2026:5460515. doi: 10.1155/jt/5460515 (PMC13343313; doi:10.1155/jt/5460515)
Supplement: Supplementary file 1 — Supporting Information Additional supporting information can be found online in the Supporting Information section. [file JT-2026-5460515-s001.pdf]

Supplementary Table 1 (A). Sample size calculation for adult *Drosophila melanogaster* flies using G*Power analysis

**F tests** - ANOVA: Fixed effects, omnibus, one-way			
**Analysis:**	A priori: Compute required sample size		
**Input:**	Effect size f	=	.70
	α err prob	=	0.05
	Power (1-β err prob)	=	0.80
	Number of groups	=	3
**Output:**	Noncentrality parameter λ	=	11.7600000
	Critical F	=	3.4668001
	Numerator df	=	2
	Denominator df	=	21
	Total sample size	=	24
	Actual power	=	0.8217701

Total Sample= 24

No. Drosophila= 8 Flies/group

Supplementary 1 (B). Sample size calculation in *Drosophila melanogaster* eggs for a Developmental experiment using G*Power analysis.

**F tests** - ANOVA: Fixed effects, omnibus, one-way			
**Analysis:**	A priori: Compute required sample size		
**Input:**	Effect size f	=	.25
	α err prob	=	0.05
	Power (1-β err prob)	=	.95
	Number of groups	=	3
**Output:**	Noncentrality parameter λ	=	15.7500000
	Critical F	=	3.0320649
	Numerator df	=	2
	Denominator df	=	249
	Total sample size	=	252
	Actual power	=	0.9514888

Total Sample= 252

84 Eggs/ group
